# Successful Surgical Management of Omental Lymphangioma in an Adult: A Case Report

**DOI:** 10.7759/cureus.84331

**Published:** 2025-05-18

**Authors:** Ruba H Aldhaheri, Norah I Alabdulaaly, Saad H Aldosari, Khaled H Altoukhi, Sarah S Alobaid

**Affiliations:** 1 General Surgery, Prince Sultan Military Medical City, Riyadh, SAU; 2 Surgical Oncology, Prince Sultan Military Medical City, Riyadh, SAU

**Keywords:** adult lymphangioma, gastro-intestinal surgery, midline laparotomy, omental cyst, omental lymphangioma

## Abstract

Lymphangioma is an uncommon benign lesion typically observed in childhood, with rare occurrences in adults. The preferred treatment for this condition is surgical resection, which offers an excellent prognosis and minimal risk of recurrence when complete removal is achieved. This case report describes the successful surgical management of a rare omental lymphangioma in a 26-year-old man who presented with epigastric pain and abdominal distension. Diagnostic imaging revealed a massive multiloculated cystic lesion measuring 9.0 × 21.3 × 26 cm occupying the entire omentum, with evidence of partial rupture and hemorrhagic fluid. The patient underwent complete surgical excision via midline laparotomy, which required partial gastrectomy due to tumor adherence to the stomach. Histopathological examination confirmed the diagnosis of lymphangioma, and the patient recovered well with no recurrence at 12-month follow-up. This case highlights the diagnostic challenges of adult-onset abdominal lymphangiomas and underscores the importance of complete surgical resection as the definitive treatment. Large omental lymphangiomas can present significant surgical challenges and require careful preoperative planning with advanced imaging and consideration of adjacent organ involvement.

## Introduction

Cystic lymphangioma is a benign proliferation of ectopic lymphatic tissue that typically does not communicate with the normal lymphatic system [1]. While most commonly occurring in the cervical region of children, abdominal cystic lymphangiomas are rare, accounting for less than 5% of all cases [2]. In adults, their incidence is exceptionally low, estimated at 1 in 100,000 to 1 in 250,000 [1]. The pathogenesis remains unclear, but proposed mechanisms include developmental failure of lymphatic channels, inflammatory obstruction, or degeneration of lymph nodes [3]. Due to their nonspecific presentation, which can range from asymptomatic incidental findings to acute abdominal pain or distension, the diagnosis is often challenging and relies heavily on imaging modalities such as ultrasound, computed tomography scan (CT), and magnetic resonance imaging (MRI) [4, 5].

The management of abdominal lymphangiomas is particularly complex in adults due to their potential for large size and involvement of critical structures. As highlighted in the discussion, complications such as hemorrhage, rupture, or intestinal obstruction may occur, necessitating prompt intervention [6]. Complete surgical excision remains the treatment of choice, as incomplete resection increases the risk of recurrence [4]. However, the surgical approach must be tailored to the tumor's anatomical relationships, which may require resection of adjacent organs. We present a case of omental lymphangioma in an adult that contributes to the limited literature on adult omental lymphangiomas and reinforces the importance of preoperative imaging, multidisciplinary planning, and complete resection to ensure optimal outcomes.

## Case presentation

A 26-year-old man presented to the emergency department with epigastric abdominal pain lasting for five days. The pain began gradually and did not radiate, and was associated with nausea and vomiting. The patient denied any history of similar complaints, fever, changes in bowel movements, or urinary symptoms. Upon examination, he appeared well, was not in severe pain or distress, and had a normal body build. The abdominal examination revealed mild distention, with a palpable mass in the epigastric area extending to the paraumbilical region. There were no skin changes or signs of peritonitis. Routine blood investigations showed no obvious abnormalities. An abdominal X-ray was unremarkable.

**Figure 1:**
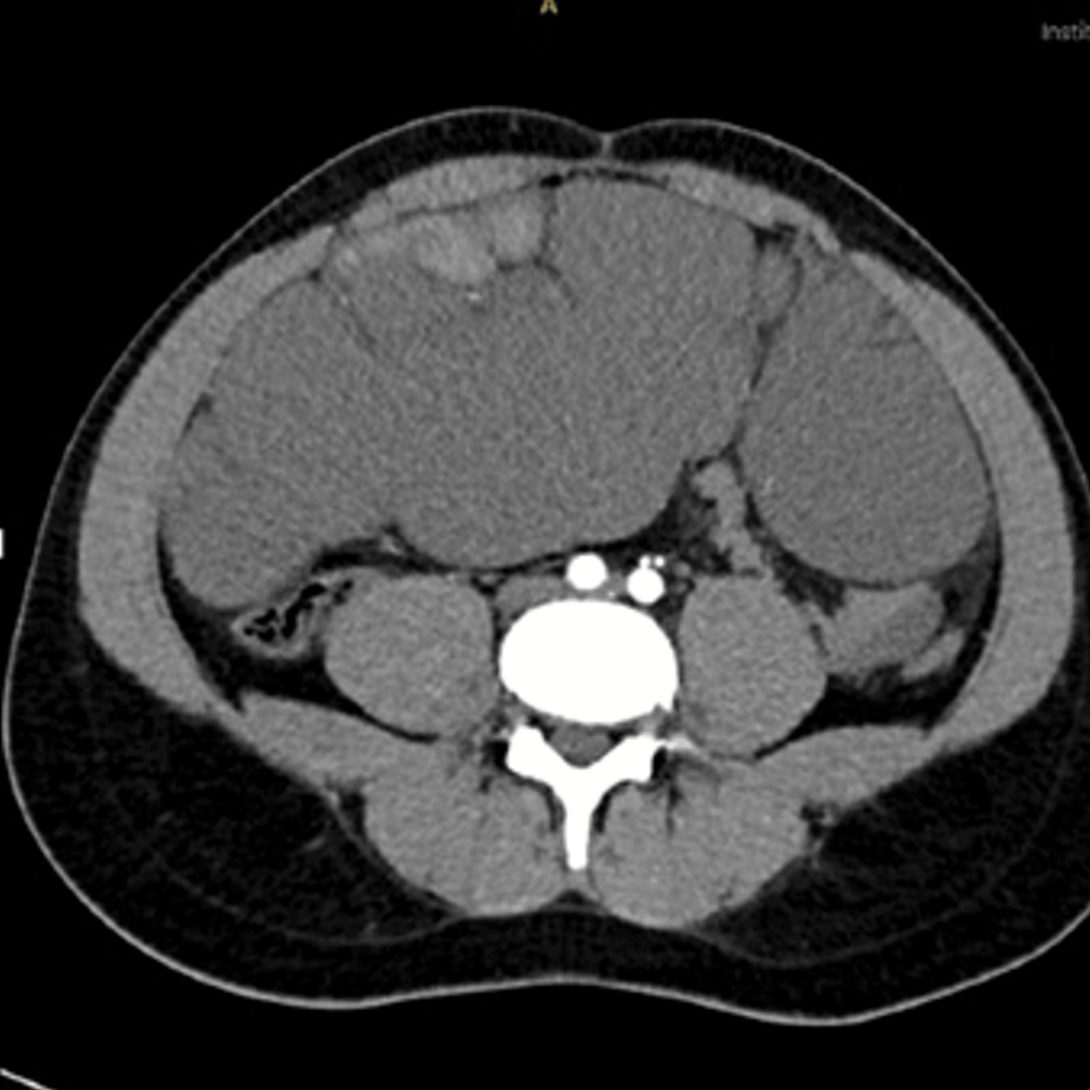
Abdominal computed tomography (CT) showing multiloculated cystic structures filling the entire omentum, and displacing the bowel loops posteriorly

**Figure 2:**
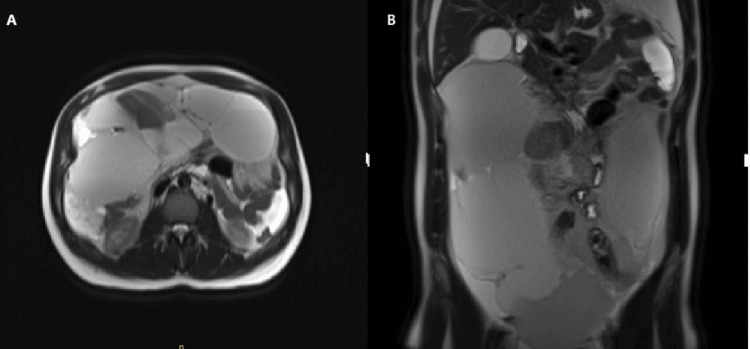
An MRI transverse (A) and coronal (B) sections of the abdomen showed a multiloculated cystic lesion measuring approximately 9.0 x 21.3 x 26 cm

Abdominal computed tomography (CT) revealed multiloculated cystic structures filling the entire omentum, extending into the rest of the peritoneal cavity and displacing the bowel loops posteriorly, with no associated aggressive features (Figure 1). An MRI of the abdomen showed a multiloculated cystic lesion measuring approximately 9.0 x 21.3 x 26 cm, with hematomas, the largest of which was located in the right anterolateral aspect of the peritoneal cavity, measuring 10 x 4.8 cm. There was evidence suggestive of partial rupture, with the development of a moderate amount of free hemorrhagic fluid in the peritoneal cavity (Figure 2).

**Figure 3:**
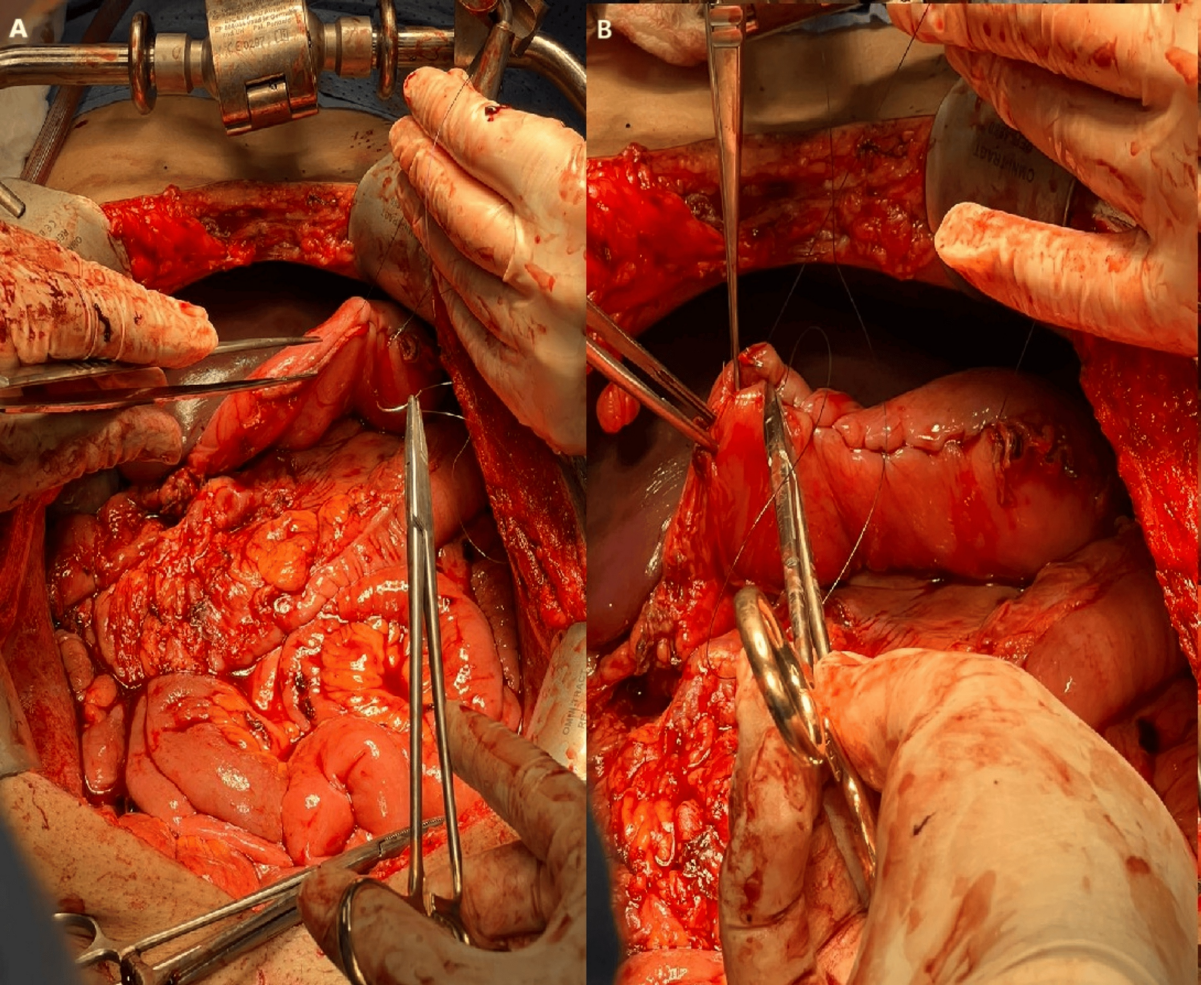
Complete excision with partial gastrectomy was performed and sutures of the stomach (A and B)

The case was discussed with the patient, and a management plan was proposed, with an agreement to proceed with surgical resection. A midline laparotomy was performed, revealing a large cyst that was inseparable from the greater curvature of the stomach. Due to the uncertainty of the diagnosis and the inability to rule out malignancy, the decision was made to perform a partial gastrectomy to achieve R0 resection. Complete excision was successfully carried out (Figure 3).

The postoperative period was uneventful, and the patient was discharged home in good condition on postoperative Day 8. A dietitian was involved in postoperative dietary management after partial gastrectomy. The patient was seen in the clinic one week after discharge and reported doing well, with no active or new complaints. Histopathological analysis revealed a multilocular cyst with dilated lymphatic spaces lined by flat endothelium, consistent with lymphangioma. The gastric mucosa showed chronic active gastritis with *Helicobacter pylori *(*H. pylori*) infection. Eradication therapy for *H. pylori *was administered. No evidence of recurrence was found during the follow-up period, which extended to 12 months.

## Discussion

Cystic lymphangioma is a rare benign vascular malformation consisting of thin-walled cysts [4]. The etiology of omental lymphangioma remains unclear, with several theories suggesting possible causes, including trauma, failure of connection between embryonic channels and the venous system, trauma, inadequate fusion of the mesenteric leaves, and degeneration of lymph nodes [5].

Cystic lymphangioma presentations are often nonspecific and can vary depending on the involved site, making diagnosis challenging. Patients can present with acute symptoms, such as acute abdominal pain, fever, distension, and vomiting, and fever. Chronic symptoms often manifest as progressive abdominal distension and discomfort [6, 7].

Complications can include small bowel obstruction, intestinal volvulus, or infarction; furthermore, cyst complications include hemorrhage, infection, rupture, and torsion [8]. Imaging studies are essential for identifying the tumor, with abdominal ultrasound being the initial modality of choice. Ultrasound is used to estimate the cyst size, location, boundaries, and contents [9]. However, ultrasound interpretation can be complicated with hemorrhage and necrosis within the capsule, leading to changes in echogenicity.

Abdominal-enhanced CT is preferred for further evaluation, as it allows assessment of the tumor's density and its relationship with the surrounding blood vessels and organs; therefore, it differentiates retroperitoneal from intraperitoneal lymphangioma. MRI is useful because of its high sensitivity in detecting cystic complications. The definitive diagnosis is confirmed histopathologically, revealing abnormally dilated lymphatic vessels lined with flat endothelial cells [9-11].

The management of our 26-year-old patient with a giant omental lymphangioma aligns with and expands upon several key findings from prior literature. Like the cases reported by Maghrebi et al. [2], our patient presented with nonspecific abdominal symptoms, reinforcing that adult lymphangiomas often manifest with vague complaints that can delay diagnosis. The massive size of our lesion (26 cm) exceeded most dimensions reported in Kumar et al.'s [5] series of omental cysts.

Our surgical approach of complete excision with partial gastrectomy echoes Rao et al.'s [6] emphasis on radical resection when adjacent organ involvement occurs. The need for gastric resection in our case, due to inseparable adherence to the greater curvature, underscores a technical challenge not fully addressed in previous reports. This finding suggests that even benign lymphangiomas may exhibit aggressive local behavior in adults, warranting more extensive procedures than typically required in pediatric cases.

The excellent postoperative outcome at 12-month follow-up supports existing evidence that complete resection prevents recurrence [2]. However, our incidental discovery of *H. pylori* gastritis introduces a novel consideration - whether chronic inflammation could contribute to lymphangioma development or growth in adults, a hypothesis not previously explored in the literature. This observation merits further investigation through larger case-control studies, as it is known that lymphangiomas are developmental malformations.

These findings collectively advance our understanding of adult abdominal lymphangiomas by demonstrating their potential for extreme dimensions requiring adapted surgical strategies, highlighting unique diagnostic pitfalls in adults compared to pediatric presentations, and introducing possible inflammatory etiologic factors.

The case thus bridges gaps between prior reports while identifying new directions for research on this rare condition.

Complete surgical excision is the treatment of choice, with an excellent prognosis associated with complete resection, whereas incomplete resection may lead to recurrence [12]. Total surgical excision is regarded as the most effective treatment strategy. Achieving complete removal of the affected tissue greatly enhances the likelihood of a favorable outcome for the patient. When the entire mass, along with a margin of healthy tissue, is successfully excised, the risk of recurrence is minimized, leading to better long-term health prospects.

Conversely, when only a partial resection is performed, the chances of residual disease increase, which can result in a return of the condition. This necessitates further medical intervention, which may involve additional surgeries.

## Conclusions

The successful surgical management of this rare omental lymphangioma case highlights key clinical insights for adult presentations of this typically pediatric condition. The patient's nonspecific symptoms and the tumor's large size with hemorrhagic rupture underscore both the diagnostic challenge and the critical role of advanced imaging. CT and MRI were indispensable for characterizing the multiloculated cyst's anatomy and complications, guiding the decision for open resection with partial gastrectomy to achieve complete excision. This approach resulted in an excellent outcome, reinforcing surgical resection as the gold standard while demonstrating the need for intraoperative adaptability when tumors involve adjacent structures.

Long-term follow-up remains essential despite the benign nature of lymphangiomas, as incomplete resection increases recurrence risk. This case contributes to the limited literature on adult omental lymphangiomas and emphasizes multidisciplinary collaboration from diagnosis through postoperative care. Future research should investigate the etiology of adult-onset cases and explore minimally invasive techniques, while clinical registries could improve understanding of optimal management strategies for these rare lesions. The patient's 12-month recurrence-free outcome supports complete surgical excision as curative, but continued monitoring is warranted given documented cases of late recurrence.
